# A Compendium on the NIST Radionuclidic Assays of the Massic Activity of ^63^Ni and ^55^Fe Solutions Used for an International Intercomparison of Liquid Scintillation Spectrometry Techniques

**DOI:** 10.6028/jres.102.035

**Published:** 1997

**Authors:** R. Collé, B. E. Zimmerman

**Affiliations:** National Institute of Standards and Technology, Gaithersburg, MD 20899-0001

**Keywords:** beta decay, efficiency tracing, hydrogen-3, intercomparison, iron-55, liquid scintillation (LS), measurements, nickel-63, radioactivity, spectrometry

## Abstract

The National Institute of Standards and Technology recently participated in an international measurement intercomparison for ^63^Ni and ^55^Fe, which was conducted amongst principal national radionuclidic metrology laboratories. The intercomparison was sponsored by EUROMET, and was primarily intended to evaluate the capabilities of liquid scintillation (LS) spectrometry techniques for standardizing nuclides that decay by low-energy *β*-emission (like ^63^Ni) and by low-*Z* (atomic number) electron capture (like ^55^Fe). The intercomparison findings exhibit a very good agreement for ^63^Ni amongst the various participating laboratories, including that for NIST, which suggests that the presently invoked LS methodologies are very capable of providing internationally-compatible standardizations for low-energy *β*-emitters. The results for ^55^Fe are in considerably poorer agreement, and demonstrated the existence of several unresolved problems. It has thus become apparent that there is a need for the various international laboratories to conduct rigorous, systematic evaluations of their LS capabilities in assaying radionuclides that decay by low-*Z* electron capture.

## 1. Preliminary Notes

### 1.1 Radionuclidic Metrology for *β*– and EC Decay Modes Having Low-Energy Radiations

Determinations of the activity for nuclides that decay by pure, low-energy *β*–emission and low-*Z* (atomic number) electron capture (EC) to the ground state of their daughters are amongst the most difficult within the realm of radionuclidic metrology. The difficulties arise from the low-energy radiations associated with these decay modes (which are easily absorbed in counting sources and which require large scattering and absorption loss corrections), and from the absence of any *γ* rays and other coincident transitions (which precludes use of standardization techniques like *γ*-ray spectrometry or primary *β–γ* coincidence methods). In the past decade, considerable progress has been made in applying liquid scintillation (LS) spectrometry to the assay of such radionuclides through 4*π* detection of the *β* particles or the Auger electrons accompanying the EC decay.

### 1.2 ^63^Ni and ^55^Fe Decay

The nuclide ^63^Ni, with a half-life of *T* = 101.1 a ± 1.4 a [1], decays by pure *β*-emission to the ground state of ^63^Cu by an allowed transition (*J^π^* = 1/2^–^ → 3/2^–^) having a well-known *β*-spectrum maximum endpoint energy of *E_β_*_(max)_ = 66.945 keV ± 0.004 keV [2–4] and a number-weighted mean energy of *E_β_*_(mean)_ = 17.426 keV ± 0.013 keV [4].

The nuclide ^55^Fe decays by pure EC (a 231.6-keV transition) to the ground state of ^55^Mn (*J^p^* = 3/2^–^ → 5/2^–^) with the attendant plethora of x rays and Auger electrons that result from multiple shell captures (e.g., *P*_K_ = 0.88, *P*_L_ = 0.10, and *P*_M+N_ = 0.02 for the probabilities for K-shell, L-shell, and higher-shell capture [5]) followed by both radiative (x = ray) and nonradiative rearrangements (Auger effect). The numerous electron vacancy fillings from higher shells (and their subshells) and Auger rearrangements (by vacancy transfers to higher shells, as well as accompanying Coster-Kronig inner-shell rearrangements) result in well-known, but complex x-ray and electron spectra. Maximum energies of the associated K x rays and K-shell Auger electrons are banded in the range of 5 keV to 6.5 keV, while those for the L and above shells are < 0.7 keV. The ^55^Fe half-life is, surprisingly, not very well known (or at least uniformly “accepted”), having two somewhat disparate, routinely used, values that center around *T* = 997 d ± 11 d [6] and *T* = 1009.5 d ± 1.3 d [7]. The half-life disparity is not particularly significant unless large, multiple-year, decay corrections (such as that applied to the primary NIST ^55^Fe standardization mentioned in section 1.7 below) are made.

### 1.3 Utility of ^63^Ni and ^55^Fe for Intercomparison Evaluations

Both nuclides can be exceedingly useful for evaluating the performance and practice of such aforementioned and difficult, low-energy-sensitive radionuclidic standardizations. Not surprisingly then, they were the nuclides of choice for the present international measurement intercomparison of liquid scintillation (LS) standardization techniques.

### 1.4 EUROMET Intercomparison

The intercomparison was sponsored by EUROMET (a European collaboration for metrology), and organized by the Laboratoire Primaire des Rayonnements Ionisants (LPRI) of France. It was conducted in two phases: Part 1(1995) and Part 2(1996). The National Institute of Standards and Technology, although not a member of EUROMET, was invited to participate in the intercomparison (on concurrence of the member states). NIST, however, did not participate in an earlier, preliminary phase of the intercomparison which has been reported on by Cassette [8].

The participating laboratories for the intercomparison were:

BIPM: Bureau International des Poids et Mesures (Sèvres, France)
CIEMAT: Centro de Investigaciones Energeticas Medioambientales y Technologicas (Madrid, Spain)
CMI: Cesky Metrologicky Institut (Prague, Czech Republic)
ENEA: Ente per le Nuove technologie, l’Energia e l’Ambiente (Rome, Italy)
IRMM: Institute for Reference Materials and Measurements (Geel, Belgium)
LPRI: Laboratoire Primaire des Rayonnements Ionisants (Saclay, France)
NAC: National Accelerator Centre (Faure, South Africa)
NIST: National Institute of Standards and Technology (Gaithersburg, MD, USA)
PTB: Physikalisch Technische Bundesanstalt (Braunschweig, Germany)
RC: Radioisotope Centre (Swierk, Poland)
SCK/CEN: Studie Centrum voor Kernenergie/Centre d’etude de l’Energie Nucliaire (Mol, Belgium)

### 1.5 This Compendium’s Objectives

This compendium summarizes the NIST activities and measurement results for the massic activities of ^63^Ni and ^55^Fe for the two solutions that were distributed for the final phase (Part 2) of the intercomparison. The primary objectives of this summary are to suitably archive the NIST results for this important intercomparison, and to document the experimental methodologies used to obtain the results.

The intercomparison findings *(vis-à-vis* the NIST results and those of other laboratories), as presented herein (Sec. 5), are based on a summary report by Cassette [9] and on a forthcoming paper by Cassette [10] to be presented at the 1997 meeting of the International Committee of Radionuclide Metrology.

### 1.6 Previous International Intercomparisons of ^63^Ni and ^55^Fe

Measurement intercomparisons of ^63^Ni and ^55^Fe among national metrological laboratories have been previously performed.

A NIST microcalorimetry-based standardization of ^63^Ni (performed in 1968) [11, 12] was informally inter-compared by three sister national metrology laboratories with their own standardizations [13–15], over the following 3 years, using 4*πβ* coincidence efficiency-tracing methods (see references therein). The agreement with the NIST calorimetry-based standardization was within a few percent for all three laboratories. These intercomparison results have been summarized by Barnes et al. [11], by Lowenthal, et al. [15], by Coursey et al. [16], and by Mann and Unterweger [17]. Collé and Zimmerman [18], more recently, re-evaluated (with more appropriate data re-normalizations) these earlier ^63^Ni intercomparison findings.

A more formal international intercomparison of ^55^Fe measurements was organized by the Bureau International des Poids et Mesures (BIPM) in 1978, and has been described by Smith and Woods [19] and by Smith [20]. Eleven laboratories, including NIST, participated in the exercise. These various laboratories performed either measurements of the massic activity or massic K x-ray emission rate (or both) of a ^55^Fe solution that was distributed for the purpose. The assays were performed using a large variety of different measurement methods, including some LS spectrometry techniques. The spread in the results among laboratories was generally greater than 5 % [20].

None of the measurements for the above intercomparisons utilized the currently-in-vogue LS spectrometry techniques, such as the CIEMAT/NIST efficiency-tracing protocol (described in section 2.2 below) or triple-to-double coincidence methods. Collé and Zimmerman [18], however, have recently demonstrated the excellent agreement between the 1968 calorimetry of ^63^Ni and the 1984 and 1995 assays of ^63^Ni by 4*πβ* LS spectrometry using the CIEMAT/NIST method for ^3^H-standard efficiency tracing.

### 1.7 Recent NIST Standardizations of ^63^Ni and ^55^Fe

Standardizations of ^63^Ni and ^55^Fe have also been very recently performed by NIST for issuance of Standard Reference Material SRM 4226C [21] and SRM 4929E [22].

The former, for ^63^Ni, was based on 4*πβ* LS spectrometry with ^3^H-standard efficiency tracing using the CIEMAT/NIST protocol. Details of this 1995 calibration (and corollary studies) have been given at length by Collé and Zimmerman [1, 18] and by Zimmerman and Collé [23, 24].

The latter, for ^55^Fe, was based on a decay-corrected calibration that was performed in the mid-1980s by defined solid-angle x-ray spectrometry using a thin-window NaI(Tl) detector [25]; and on 1995 confirmatory measurements by LS spectrometry using the CIEMAT/NIST method of efficiency tracing with standards of ^3^H, ^51^Cr, ^54^Mn, and ^65^Zn [26].

## 2. Overview of the ^63^Ni and ^55^Fe Assays

### 2.1 Basis of ^63^Ni Assay

Assay of the EUROMET ^63^Ni solution was based on 4*πβ* LS spectrometry using the CIEMAT/NIST method for ^3^H-standard efficiency tracing. The measurements were performed by tracing with both a NIST ^3^H standard and a LPRI ^3^H standard (see following Secs. 3.3 and 4.1). A re-assay of a NIST ^63^Ni standard (SRM 4226C) was performed concurrently. The simultaneous LS measurements also allowed tracing of the EU-ROMET ^63^Ni solution against the NIST ^63^Ni standard, which thereby could confirm the internal consistency of the tracing calculations.

Collé and Zimmerman [1, 18] and Zimmerman and Collé [23, 24] recently made extensive evaluations of cocktail stability and cocktail composition effects for the assay of ^63^Ni by the CIEMAT/NIST method. The findings of those evaluations were applied to this work. They found that cocktails prepared with typical ^63^Ni solutions (1 mol · L^–1^ HCl and Ni^+2^ carrier cations with mass fractions of up to 100 μg of Ni^+2^ per gram of solution) in any one of four commercially-prepared scintillants (nearly irrespective of the total HCl or Ni^+2^ loading in the cocktails) lead to unstable cocktails and unreliable assay results unless the cocktails contained a sufficiently high H_2_O mass fraction (greater than a few percent).

More recent studies by Collé et al. [27–29] discerned an important distinction between “cocktail stability” and what has been termed “cocktail tractability” [28], and also found that the previously observed H_2_O mass fraction dependencies were strongly correlated to the ionic content of the cocktails [29]. Their findings and the earlier H_2_O mass fraction effects [1,18,23] appear to be at variance with the cocktail composition effects reported on by Cassette [9, 10].

### 2.2 CIEMAT/NIST Efficiency Tracing Methodology

This protocol [30], originated by the Centro de Investigaciones Energeticas, Medioambientales y Technologicas (CIEMAT) and the NIST laboratories, is one of the more commonly invoked methodologies for LS spectrometry efficiency tracing. The method utilizes various updated and revised versions of the CIEMAT-developed EFFY code [31–33] to determine the detection efficiencies for cocktails of the traced radionuclide (under known and varying quench conditions) by following the experimentally-determined efficiencies for closely-matched cocktails of a ^3^H standard. Tritium (^3^H) is recommended to serve as the matched (in terms of cocktail composition and quenching) standard since extrapolations to the low-energy portions of the *β* spectra are more sensitive than that obtained with higher-energy *β*-emitting standards, e.g., ^14^C [16, 34]. The critical importance of cocktail matching, particularly for tracing low-energy *β* emitters like ^63^Ni, has been treated by Collé [35]. The methodology has been described in numerous publications by Coursey et al. [16, 34, 36, 37]. Details on its practical application, as recently invoked for ^63^Ni and considered here, are given by Collé and Zimmerman [1, 18], by Zimmerman and Collé [23, 24], and by Collé [35].

The EFFY code (described simplistically), for a given *β* emitter: first calculates a set of definitionally-assumed, energy-dependent efficiencies *ϵ*(*E*) as a function of a defined “figure of merit” *M* for given *β*-particle energies; evaluates the number distribution (by energy) of the *β* particles for that particular radionuclide (i.e., the shape of the *β* spectrum) by using the well-known differential Fermi distribution function *F*(*Z*,*E*)d*E* (with appropriate shape factor corrections) for that radionuclide; and thereby derives an overall detection efficiency *ϵ* by weighting over the entire *β* spectrum. Refer to the CIEMAT/NIST papers [30–37] for details.

In the CIEMAT/NIST method, experimentally-determined ^3^H-standard efficiencies ϵ_s_ are related to the EFFY-code generated “figures of merit” *M* for those ϵ_s_ efficiencies. They have a functional form *ϵ*_s_ = *F*_1_[*M*]. The parameter *M* (see references for definition) is used to characterize the quenching and overall detection efficiency of the LS counting system (cocktails plus spectrometer). These same *M* values are then related to similarly calculated efficiencies *ϵ*_x_ for the traced radionuclide (normally after applying a quench adjustment) with functional form *ϵ*_x_ = *F*_4_[*M*]. This is the method’s procedure at its simplest conceptual level. In effect, the two *F*_1_ and *F*_4_ functions operate like a kind of universal quench correction curve that accounts for differences in the detected portions of the *β* spectra for the ^3^H standard and traced radionuclide.

In practice, one usually prepares a set of nearly identical ^3^H cocktails and another nearly identical set of traced radionuclide cocktails, and varies the quenching (and hence the efficiencies over some range) within both sets by the controlled addition of some imposed chemical quenching agent like CCl_4_ or CH_3_NO_2_. Quenching differences due to slight cocktail mismatches are accounted for by making adjustments with experimentally-determined quench indicating parameters (QIP), such as the classical Horrocks number *H* [38]. The adjustments are made by first relating the experimentally-observed QIP values, *H*_s_, for the set of ^3^H-standard cocktails (with known detection efficiencies) to *M* values through the combined use of the relations between *H*_s_ and *ϵ*_s_ and *M* (i.e., the *ϵ*_s_ = *F*_2_[*H*_s_] and the *M* = *F*_1_[*ϵ*_s_] functions) to obtain a new relation *M* = *F*_3_[*H*_s_]. To do the QIP adjustment, one assumes that the *M* = *F*_3_[*H*_s_] function is valid for any other traced radionuclide irrespective of the differences in the underlying *β* spectra and in the sources of quenching. This is a *critical assumption*, as recently clarified by Collé [35]. The parameter *M* in the CIEMAT/NIST model can adequately account for differences in *β* spectra, detection thresholds, and for quench differences when the quenching results from the same causal factor. It can not fully adjust for quench changes caused by different agents (e.g., those due to simultaneous introduction of differences in cocktail sizes, differences in cocktail constituent components, and differences in cocktail composition concentrations) [35].

### 2.3 Basis of ^55^Fe Assay

Assay of the EUROMET ^55^Fe solution was primarily based on comparative measurements (with QIP-adjusted quench corrections) against NIST SRM 4929E for ^55^Fe. The EUROMET ^55^Fe solution was also traced against the NIST ^55^Fe standard by the CIEMAT/NIST method using the CIEMAT-developed EMI code [39, 40].

The EMI code was developed for performing efficiency calculations for nuclides that decay by internal conversion and electron capture, and its use is operationally similar to that invoked for the EFFY code. As for the EFFY code, EMI calculates overall efficiencies for a given nuclide as a function of some defined parameter, called, in this case, the “free parameter” *P*.

The “figure of merit” *M* from EFFY and the “free parameter” *P* from EMI are said to be comparable [i.e., that they represent the same physical concept in the formulization of the detection efficiencies *ϵ*(*E*)], which would presumably allow joint use of both codes for tracing ^55^Fe (with calculated efficiencies versus *P* from EMI) against a ^3^H standard (with calculated efficiencies versus *M* from EFFY) [40]. Tracing of the EUROMET ^55^Fe solution against a NIST ^3^H standard was therefore also attempted in this way. The attempts, however, were not successful (Sec. 4.3) and lead to what are believed to be unreliable results.

### 2.4 Nuclear and Atomic Data Used for the Inter-comparison

To avoid unnecessary normalization problems in comparing the various laboratories’ intercomparison results, the EUROMET organizers provided a “recommended” (“standard data”) set of nuclear data for ^3^H and ^63^Ni that was to be used in performing any LS detection efficiency calculations. This data set is summarized in Table 1.

Table 1 also contains a summary of the nuclear data actually used by NIST. As indicated, the “recommended values” for the ^63^Ni half-life *T* (used for decay corrections) and the *β* spectrum *E_β_*_(max)_ (used for the EFFY code calculations) were not employed by NIST. Their use was believed to be wholly untenable, given the recent critical evaluations of Collé and Zimmerman [18]. More importantly, use of the “recommended” values (for mere consistency) would have placed the NIST results for the EUROMET intercomparison at substantial variance with the recent NIST ^63^Ni standardizations. This might have easily lead to many future confusions and misunderstandings. Instead, estimates are provided (Sec. 4.1) of what the NIST results for the ^63^Ni massic activity *would be* if the “recommended” data are used.

The EUROMET organizers also provided a “recommended” data set of nuclear and atomic parameters for ^55^Fe decay (Table 2). The EMI-code efficiency calculations performed by NIST for the ^55^Fe efficiency tracings against the NIST ^55^Fe and ^3^H standards were largely based on use of this “recommended” set as given in Table 2. As part of the data set, the organizers also gave some relevant physical parameters for one commercial scintillant (Ultima Gold^1^, see Sec. 3.2). These included the density, average atomic-number to mass ratio, ionization potential, and absorption probabilities for the principal Mn x rays. The NIST results were based on the EMI-code default parameters for this scintillant.

### 2.5 General Schema

Both of the above assays were performed using two LS spectrometers having different operating characteristics, used several different commercially-prepared scintillants, and involved a substantial variety of cocktail preparations. The work consisted of a detailed and complex experimental design involving: the gravimetric preparation of 9 distinct series of cocktails having a total of over 215 separate cocktails; about 2100 individual LS counting measurements (i.e., about 30 d of livetime counting) for 19 distinct experimental trials; and multiple analyses (2 to 5) of the LS counting results (such as for efficiency tracings against different standards using the same simultaneously-obtained sets of counting data) for any given experiment. Data analyses alone required approximately 600 man-hours of effort.

The cocktail preparations and measurements were performed over the time intervals 6 December 1995 to 20 January 1996 for the ^63^Ni assay, and 12 February 1996 to 9 March 1996 for the ^55^Fe assay.

All of the results are reported with respect to a reference time of 1200 UT 1 January 1996.

## 3. Experimental Aspects

### 3.1 LS Spectrometers

The principal characteristics of the two spectrometers employed by NIST for the intercomparison are summarized in Table 3. The relative performance of the instruments for select measurements has been reported previously [23, 41–47].

As indicated in Table 3, a considerable number of the characteristics are common, e.g., operating mode, photomultiplier tube properties, operating temperature, and livetime determination method. Yet, some operating characteristics are clearly different, e.g., logarithmic versus linear pulse amplification (with attendant gain conversion differences), variable versus fixed pulse resolving times, and different QIP determination methods. Differences in the timing characteristics (coincidence resolving times and sum-coincident pulse resolving times) are particularly addressed by Collé, et al. [42].

Invariably, system P has a slightly larger detection efficiency than system B on comparisons of identical cocktails containing radionuclides with energy-dependent efficiencies, such as for ^3^H [23], ^63^Ni [23], ^36^Cl [41], ^205m^Pb (^209^Po daughter) [42], and ^117m^Sn [44]. The detection efficiency of the two systems for high-energy *β* emitters and *α* emitters are virtually invariant [43, 45–46].

The detection thresholds listed in Table 3 for the two LS systems are *very*, very nominal. Energy calibrations, based on peak channel locations of known transition energies (such as for the 2.3 keV conversion electrons in ^205m^Pb [42], K-shell-vacancy Auger electrons in ^55^Fe decay [this work], ^117m^Sn conversion electrons [44], as well as conversion electrons in ^99m^Tc decay and ^3^H and ^63^Ni *β* spectra endpoint energies [this work]), typically result in an extrapolated energy for channel 0 that is within about ± 1 keV (in both spectrometer systems). Furthermore, the apparent-energy extrapolations are usually lower with system B despite the fact that system B invariably has a lower overall detection efficiency than that for system P. This suggests that the apparent efficiency differences between the two systems are more the result of an artifact in the two systems’ electronics; for example, either the systems’ respective timing differences (e.g., for the formation of the coincidence gate) or some imposed pulse discrimination setting.

The QIP employed by system B is an internally-derived Horrocks number *H* which is based on the downward spectrum shift of the Compton edge of the external ^137^Cs *γ*-ray standard with increasing quenching in the cocktail. The parameter *H* corresponds to the spectral channel number shift between the quenched cocktail and an unquenched blank reference cocktail. The channel number shift *H* = (*c*_2_ – *c*_1_) is, because of the logarithmic pulse amplification, proportional to the logarithmic energy ratio *H* ~ log(*E*_2_/*E*_1_).

The internally-derived QIP obtained with system P is the parameter *tSIE*, which is based on a proprietary mathematical transform [48] of the energy distribution of the ^133^Ba generated Compton spectrum (and which is presumably related to the mean energy of the displaced Compton spectrum). The transform is said to be used to correct for spectral distortions arising, for example, from wall effects, volume variations, and color quenching. The parameter consists of a relative, decreasing quenching scale in which “unquenched” cocktails correspond to *tSIE* = 1000.

Additionally, the locations of the external *γ*-ray sources used for the QIP determinations differ in a most important regard. In system B, the ^137^Cs source is located to the side of the LS vial, whereas the ^133^Ba source in system P is located at the underside of the LS vial. As a result, QIP determinations with system P can more adequately account for drastic volume changes (since changes in the subtended solid angle between the *γ*-ray source and the contained cocktail in the vial are small for even rather large volume changes). QIP determinations with system B are more sensitive to any cocktail volume changes (due to the rapidly decreasing solid angle with decreasing volumes). Alternatively, QIP determinations with system B have substantially greater reproducibility in measuring multiple cocktails of nearly identical quenching (i.e., composition). The precision of QIP determinations with system P, which produces the Compton spectra with transmission of the *γ* rays through the highly irregular, excess glass at the bottoms of LS vials, is decidedly poorer.

### 3.2 Scintillants

Table 4 summarizes the scintillants used for this work. These commercially-prepared fluids contain a complex mix of a principal solvent, scintillation fluors, various surfactants and emulsifiers, chemical waveshifters, etc. Only two of the listed scintillants (UG and PCS) were used for the preparation of cocktails of the EUROMET solutions of ^63^Ni and ^55^Fe, although the other two (RS and IG) were used for preliminary studies of cocktail composition effects [23, 24]. The composition information given is that as reported by the respective manufacturers, and lists the principal solvent first and the scintillation fluor last.

### 3.3 Radionuclidic Solutions

A summary of the various radionuclidic solutions used by NIST for the intercomparison is given in Table 5. It includes the EUROMET solutions as well as the tracing and comparative-measurement standards.

### 3.4 Cocktails

Nine series of cocktails, with varying compositions, were prepared for the intercomparison measurements: five for ^63^Ni and four for ^55^Fe. The cocktail compositions are summarized in Tables 6 and 7, and are characterized in terms of the following parameters: total cocktail mass *m* (in units of g); H_2_O mass fraction *f*_w_ in the cocktail; the HCl concentration *c*_HCl_ (in units of mol · L^–1^) in the aqueous portion of the cocktail; total mass of the Ni^+2^ or Fe^+3^ cations *m*_Ni_ or *m*_Fe_ (in units of μg) in the cocktail. To vary the efficiencies of cocktails within a given series, each cocktail also contained a variable quantity (0 mg to 200 mg) of a 10 % solution of CH_3_NO_2_ in ethanol (by volume) as an imposed chemical quenching agent. The additions within a series of usually seven cocktails had nominal 10 mg to 15 mg increments in added CH_3_NO_2_ solution mass.

The first series of cocktails (A in Table 6) for ^63^Ni employed use of an EDTA^–2^ (ethylenediaminetetra-acetate) chelating agent and a relatively high H_2_O mass fraction *f*_w_. This trial was made because of a previous report of the benefits of Ni^+2^ chelation in LS assays of ^63^Ni. Slight increases in detection efficiencies and substantial, factor-of-10 improvements in measurement precision, were reported [49]. The second series (B) for ^63^Ni was similar to that for series A, except that the cocktails were prepared without the EDTA^–2^. The conditions for this series were those previously found to be most reliable for the ^3^H-standard efficiency tracing of ^63^Ni by the CIEMAT/NIST method [18, 23]. Based on the previous ^63^Ni investigations by NIST [18, 23], it was believed (a priori) that the series C cocktails with very low *f*_w_ would lead to faulty efficiency tracing results. Nevertheless, the series was included since we believed that it was likely that some other laboratories would use similar low-*f*_w_ cocktail compositions for the intercomparison, and that it would be useful to have direct comparative data. The organizers of the EUROMET inter-comparison in their supplementary information to the participants clearly stated that use of 25 mg to 100 mg of the EUROMET ^63^Ni solution in UG scintillant would result in cocktails that would be stable for at least 2 months (but that solutions with 80 μg Ni^+2^ per gram of solution would result in unstable cocktails). One might then infer that the EUROMET organizers’ considered that such low-*f*_w_ cocktails would lead to reliable efficiency tracing results. This is an inference in conflict with the previous NIST work [18, 23]. The final two cocktail series (D and E) for ^63^Ni had compositions similar to that for series B and C, except that an alternative scintillant (PCS) was used. This scintillant is no longer commercially available, but was secured from a cache stored by NIST for the past several years (for just such special occasions!) [47]. This xylene-based scintillant was chosen to examine possible differences in the efficiency tracing that might result from its use compared to the use of the newer “environmentally safe” (i.e., non-toxic, non-flammable, and bio-degradable) scintillants like UG. Most of the initial development work on the CIEMAT/NIST tracing method was performed with very stable cocktails that used the older, “environmentally unsafe” scintillants [16, 34, 36, 37].

For the ^55^Fe cocktails (Table 7), the first series (F) had a very low *f*_w_. The two sets of cocktails within this series were only intended to be used to perform a direct comparative measurement between the EUROMET ^55^Fe solution and the NIST ^55^Fe standard. These cocktails, as for the comparable ^63^Ni series, were only prepared to have a reference basis for possible future comparisons with the results of other laboratories. It was also believed (*a priori*) that their use would result in unreliable assay results because of the absence of a sufficiently high *f*_w_. The second ^55^Fe series (G) contained high H_2_O fractions. This series was prepared not only to make direct comparative measurements of the EUROMET ^55^Fe solution against the NIST ^55^Fe standard, but also to attempt to efficiency trace the EUROMET ^55^Fe solution against a NIST ^3^H standard using the CIEMAT/NIST methodology. The samples XW1 through XW7 in series G were prepared to perform a classical standard-addition experiment in which the cocktails contained known masses of both the unknown EUROMET ^55^Fe solution and known NIST ^55^Fe standard (and thereby also more closely match the Fe^+3^ compositions in the two sets of cocktails). The remaining two series of cocktails (H and I) were prepared to track possible comparative measurement differences that might arise from differences in the Fe^+3^ cation loadings in the respective cocktails (i.e., in the EUROMET ^55^Fe solution and NIST ^55^Fe standard cocktails). The former (series H) was based on a careful gravimetric adjustment of the Fe^+3^ carrier content of the EUROMET ^55^Fe solution. The latter (series I) varied the Fe^+3^ cocktail loadings by the controlled additions of variable quantities of blank Fe^+3^ carrier to both the EUROMET ^55^Fe solution cocktails and the NIST ^55^Fe standard cocktails.

Blank cocktails of comparable composition were also prepared for each of the cocktail series, and were used for counting background subtractions.

The gravimetric sample-mass determinations and LS cocktail preparation procedures that were employed for this work (and used routinely at NIST) have been described at length previously [23, 41, 43, 47].

### 3.5 Experiments

Each of the cocktails within a given series was replicately measured on either one or the other spectrometer (or both) from 4 to 10 times. Counting time intervals on each cocktail ranged from 15 min to 40 min. The counting sources (with blanks interspersed) were sequentially measured in orders (e.g., TF1, F1, TN1, N1, TF2, F2, TN2, N2, TN3, F3, TN3, N3. … for series A) such that adjacent samples were paired to those of comparable quenching. Each cocktail in any given sequence was measured once before initiation of its next replication. The replication measurements of any one cocktail were thus separated by time intervals of at least 4 or more hours.

Typical relative standard deviations of the mean for five replicate measurements (after appropriate background and decay corrections) on any one cocktail were generally less than 0.1 % (which was nearly comparable to the reproducibilty in the tracing results between cocktails in a given series).

Tables 8 and 9 summarize the various experiments used to assay the EUROMET ^63^Ni and ^55^Fe solutions. The experimental designs were tied, obviously, to the foregoing cocktail preparations. The two tables largely identify the particular experiment’s objective (in terms of, for example, what solution was traced against what standard or what the comparison basis was) as well as tabulate some of the experimental conditions (cocktails employed, spectrometer used, age of the cocktails in terms of the time interval between cocktail preparation and measurement, and efficiency and QIP ranges).

## 4. Measurement Results

### 4.1 Assay of EUROMET ^63^Ni

The NIST results for the massic activity *C*_A_(^63^Ni) of the EUROMET ^63^Ni solution was reported to be *C*_A_(^63^Ni) = 39.80 kBq · g^–1^ ± 0.16 kBq · g^–1^ (as of the reference time 1200 UT 1 January 1996). The cited uncertainty is a combined standard uncertainty (an assumed standard deviation) [50, 51] as obtained from the analysis outlined in Sec. 4.4. The reported central value for *C*_A_(^63^Ni) was largely derived from the results presented in Table 10.

The values in Table 10 were derived with the EFFY4-code conditionals given in Table 1 and discussed in Sec. 2.4. If one invokes the wholly-outdated, but “recommended” nuclear data for ^63^Ni (given in Table 1) for the efficiency tracing, then the NIST results for *C*_A_(^63^Ni) in Table 10 would increase by an average of about 0.40 % to 0.45 % (for the given efficiency ranges). The apparent massic activity *C*_A_(^63^Ni) in this case (due to just the change in the nuclear data assumptions) would be approximately 40.0 kBq · g^–1^.

**Fig. 1:**
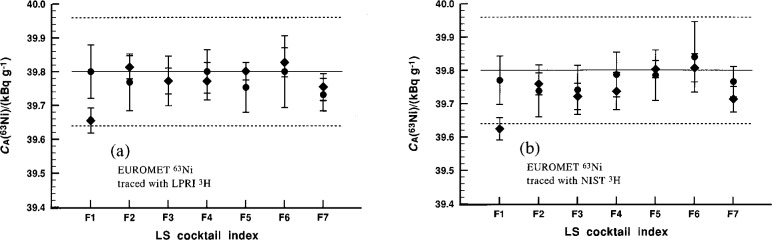
NIST results for the massic activity *C*_A_(^63^Ni) of the EUROMET ^63^Ni solution (in units of kBq · g^–1^) for seven cocktails (series A) traced against the LPRI ^3^H standard [left (a)] and the NIST ^3^H standard [right (b)] using the CIEMAT/NIST method. The closed circles and closed diamonds represent values obtained with the system B spectrometer (experiment 1) and system P spectrometer (experiment 2), respectively. The uncertainty intervals on each datum correspond to the calculated standard deviation obtained from four replicate measurements on each set of seven cocktails. The solid and broken horizontal lines correspond, respectively, to the mean *C*_A_(^63^Ni) and its combined standard uncertainty interval as reported by NIST for the intercomparison.

The quality of the tracing data may be appreciated by examination of the representative data given in Fig. 1 for one series of cocktails as measured with both spectrometers (experiments 1 and 2). As seen here, the between-cocktail variability (within a given cocktail series and experiment) is comparable to the measurement *repeatability* on any one cocktail in the series. The variability between experiments (and cocktail series), as shown in Table 10, is larger. There are in fact three distinct and evaluatable components of measurement variability in the efficiency tracing results: (1) that due to the LS measurement repeatability of the traced mas-sic activity for a given individual LS cocktail (typically 0.06 % for a relative standard deviation of the mean with υ = 4 degrees of freedom); (2) that due to the *reproducibility* among differently-quenched cocktails (of similar composition) with a single-efficiency tracing experiment (0.06 % to 0.15 % for the relative standard deviation for υ = 6); and (3) that due to the *reproducibility* between efficiency-tracing experiments with cocktails of different compositions (0.17 % for the relative standard deviation with υ = 4 or υ = 5). The magnitudes of the latter two components may be derived from the results of Table 10.

There appears to be a slight systematic difference in the results for tracing against the LPRI ^3^H standard compared to those obtained from tracing against the NIST ^3^H standard. The results for *C*_A_(^63^Ni) obtained using the LPRI standard are invariably larger. The average relative difference obtained from the first five experiments listed in Table 10 is 0.09 %. The largest relative difference in any one experiment was 0.29 %. These differences are well within the uncertainties of the ^3^H standards (Table 5), and confirm the good agreement between these two national ^3^H standards.

Evidently, on comparing the tracing results from experiments 1 and 2 versus those from experiments 3 and 4 (Table 10), the effect of EDTA^–2^ chelation was negligible. There were also virtually no significant difference for cocktails of varying age (on comparing experiments 1 and 7) and between spectrometers (on comparing experiments 1 and 3 against 2 and 4). There is some suggestion that the results obtained with the xylene-based alternative scintillant PCS (experiment 9) are low compared to that obtained with UG in the other experiments. The limited data and magnitude of the difference (about 0.4 % on a relative basis) does not, however, make this conclusion necessarily compelling. Nevertheless, the integrity of the scintillant may be questionable because of its age.

Table 11 summarizes the experimental trials for the ^63^Ni assays that were *a priori* believed to be unreliable because of the low H_2_O mass fractions *f*_w_ in the cocktails. The results largely confirm the previous findings [18, 23] on the need for a sufficiently large *f*_w_, even for cocktails containing the much lower Ni^+2^ concentration that was present in the EUROMET ^63^Ni solution.

### 4.2 Re-assay of NIST ^63^Ni

Table 12 summarizes the tracing results obtained for the re-assay of the NIST ^63^Ni standard (SRM 4426C). The decay-corrected (to the 1200 UT 1 January 1996 reference time) certified value for the C_A_(^63^Ni) massic activity of the standard is *C*_A_(^63^Ni) = 50.40 kBq · g^–1^ ± 0.24 kBq · g^–1^ [21, 23] where the uncertainty is a combined standard uncertainty (an assumed standard deviation).

The evident confirmation of the original standardization provides a comforting reassurance to the tracing work performed for this intercomparison exercise.

As before, use of the EUROMET “recommended” nuclear data for ^63^Ni would have increased the values in Table 12 by an average of about 0.40 % to 0.45 % (for the given efficiency ranges). This change would place the re-assay results nearly outside the uncertainty interval for the NIST ^63^Ni standard.

The results of Table 12 are highly correlated with those of Table 10 (being based on identical LS counting data for the two ^3^H standards). The results, not surprisingly then, exhibit the same features as those described above for the assay of the EUROMET ^63^Ni solution, *viz.*, systematically larger *C*_A_(^63^Ni) values for tracing with the LPRI ^3^H standard; absence of a significant chelation effect; invariance with employed spectrometer and cocktail ages; and a suggested significant difference with use of the PCS scintillant.

For comparisons, the *a priori* excluded experimental trials (due to low *f*_w_) for the re-assays are given in Table 13.

### 4.3 Assay of EUROMET ^55^Fe

The NIST result for the massic activity of the EUROMET ^55^Fe solution was reported to be *C*_A_(^55^Fe) = 52.95 kBq · g^–1^ ± 1.18 kBq · g^–1^ (as of the 1200 UT 1 January 1996 reference time). The result was based exclusively on direct comparative LS measurements (with QIP-adjusted quench corrections) against the NIST ^55^Fe standard (SRM 4929E) [22, 26]. The stated uncertainty of *C*_A_(^55^Fe) corresponds to a combined standard uncertainty (an assumed standard deviation) and has components dominated by the uncertainty of the NIST ^55^Fe standard (Sec. 4.4).

Table 14 summarizes the comparative measurements for five experiments that were considered to be valid (having a sufficiently high *f*_w_). The uncertainties for the between-cocktail reproducibility within a series (or within an experiment) are roughly an order of magnitude larger than those found for the ^63^Ni tracing experiments. This may be seen by comparisons of the tabulated standard deviations in Table 14 with those in Tables 10 and 12. The measurement variability (repeatability) for any one cocktail is about the same in both the ^55^Fe and ^63^Ni experiments. The large uncertainty differences are attributed to composition-dependent instabilities in the ^55^Fe cocktails (whose cause and nature is presently unknown) that result in much more variable and sensitive quench curves.

**Fig. 2:**
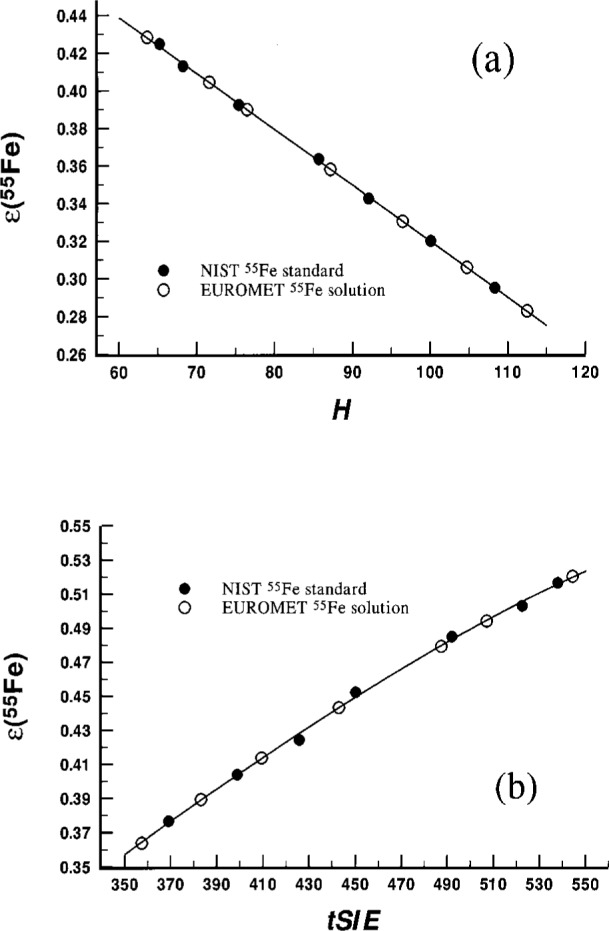
Representative quench curves (efficiency (^55^Fe) versus the H and *tSIE* QIPs) used by NIST for two of the comparative measurements of the EUROMET ^55^Fe solution. The solid circles represent data used to develop the curves from a NIST ^55^Fe standard. The open circles represent points on the curves for the matched EUROMET ^55^Fe cocktails. The upper (a) and lower (b) curves were obtained with spectrometer systems B (experiment 11) and P (experiment 15), respectively.

Figure 2 shows typical quench curves for two of the experiments.

A major concern in these comparative measurements was that although the cocktails prepared with the EUROMET ^55^Fe solution and the NIST ^55^Fe standard were reasonably matched in terms of cocktail masses *m* (or volumes), H_2_O fractions *f*_w_, and HCl concentration *c*_HCl_, there were substantial differences in their Fe^+3^ mass *m*_Fe_ (see Table 6) for some initial experiments. As demonstrated by Collé [35], this can sometimes create quench correction problems, and could invalidate the use of a quench curve developed with standards having one *m*_Fe_ value when applied to unknown solution cocktails having another m_Fe_ value. Hence, experiment 14 (using standard additions)and experiment 16 (with a Fe^+3^ carrier adjustment of the EUROMET ^55^Fe solution) were performed to account for possible quench correction errors in experiments 11, 12, and 15.

**Fig. 3:**
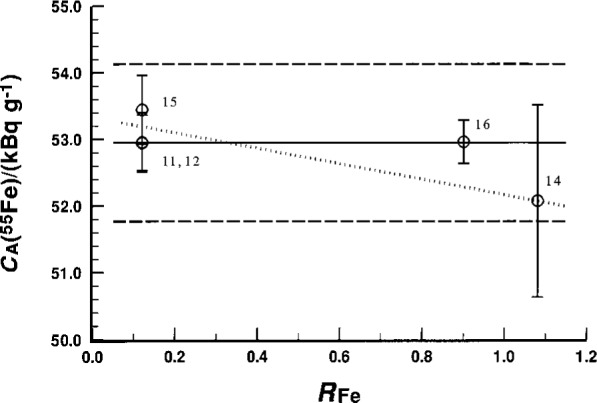
NIST results for the massic activity C_A_(^55^Fe) of the EUROMET ^55^Fe solution (in units of kBq · g^–1^) as obtained from five comparative measurements (against a NIST ^55^Fe standard). The mean values of C_A_(^55^Fe) from each experiment are given as a function of the parameter *R*_Fe_, which is the ratio of the total Fe^+3^ mass loadings *m*_Fe_ in the two sets of cocktails used for the given experiment. See the text for a discussion. The solid and broken horizontal lines correspond, respectively, to the mean *C*_A_(^55^Fe) and its combined standard uncertainty interval as reported by NIST for the intercomparison.

A useful parameter to examine this possible error is *R*_Fe_, the ratio of *m*_Fe_ in the EUROMET ^55^Fe solution cocktails to *m*_Fe_ in the NIST ^55^Fe standard cocktails. The results obtained for *C*_A_(^55^Fe) as a function of the parameter *R*_Fe_ are shown in Fig. 3. A value of *R*_Fe_ = 1 would correspond to perfect cocktail matching. Prior to the result obtained with experiment 16 (with *R*_Fe_ = 0.90), there was a suggestion of a possible *R*_Fe_ dependence because of the low *C*_A_(^55^Fe) value obtained in experiment 14 (*R*_Fe_ = 1.08). This suggested systematic dependence is exhibited by the dotted line in Fig. 3. The large uncertainty in the standard addition experiment (14), however, precludes a definite conclusion. The results of experiment 16 were in very good agreement with those of experiments 11, 12, and 15 which had *R*_Fe_ = 0.12.

Table 15 contains the results for *C*_A_(^55^Fe) for the three experiments that were not considered to be valid.

Experiment 10 was performed with low *f*_w_ and had a mean *C*_A_(^55^Fe) that was about 1 % lower than the values obtained in experiments 11 and 12. This could very well be a valid result since the small magnitude of the difference (–1.1 %) is comparable to that found for the difference (+ 0.9 %) between experiment 15 and experiments 11 and 12. Nevertheless, our prior and continuing investigations constrain us to suspect the results from any UG-based cocktails containing low *f*_w_.

Experiment 13 was an attempt to trace the EUROMET ^55^Fe solution against a NIST ^3^H standard using the CIEMAT/NIST methodology (with EMI-code calculations for the ^55^Fe efficiencies versus the free parameter *P* and with EFFY4-code calculations for the ^3^H efficiencies versus the figure of merit *M*). The traced mean *C*_A_(^55^Fe) was over 10 % low compared to any comparative measurement against the NIST ^55^Fe standard. Use of the CIEMAT/NIST method (only using the EMI code) to trace the EUROMET ^55^Fe solution against the NIST ^55^Fe standard (experiment 12) gave a result that was indistinct from that obtained from the quench-curve comparative measurement (experiment 11). This is not surprising inasmuch as the two calculational approaches used identical LS counting data, and use of the EMI code in this case merely introduced an additional sequence of quench correction steps by application of the free parameter *P*.

Experiment 17 was also based on cocktails with low *f*_w_ and was thereby excluded. The experiment was performed to investigate cocktail composition effects under variable conditions. The cocktails (series I) contained variable quantities of the ^55^Fe solutions and controlled additions of a Fe^+3^ carrier solution, such that they contained a sequence of variable *f*_w_, variable *m*_Fe_, and variable total HCl concentration *m*_HCl_ (along with a variable QIP *H*). All of the cocktails in the series contained constant *c*_HCl_ ≅ 1 mol · L^–1^ but had variable *m*_HCl_ loadings given by *m*_HCl_ = *mf*_w_*c*_HCl_/*r*, where the density *r* ≅ 1.015 g · mL^–1^ is for nominal 1 mol · L^–1^ HCl. These combinations of cocktail-component variables could be used to obtain an interesting variety of quench corrections (where the corrections are based on extrapolations against the cocktail-component variables using the cocktail-component variables themselves as a QIP), which will be reported on elsewhere [27–29]. The result given in Table 15 for experiment 17 is just that based upon a comparative measurement using the *H* QIP for quench corrections.

### 4.4 Uncertainty Analyses

A complete analysis of the measurement uncertainties for the massic activity of the EUROMET ^63^Ni solution is outlined *in extenso* in Table 16.

The uncertainty analysis procedure follows the normal conventions of the NIST Radioactivity Group which are compatible with those adopted by the principal international metrology standardization bodies [50, 51]. All individual uncertainty components, called “standard uncertainties,” are expressed in terms of estimated (experimental) standard deviations (or standard deviations of the mean where appropriate) or quantities assumed to correspond to standard deviations, irrespective of the method used to evaluate their magnitude. A propagated “combined standard uncertainty” is expressed as an equivalent standard deviation which is equal to the positive square root of the total variance obtained by summing all variance and covariance components, however evaluated, using the law of propagation of uncertainty for the specific mathematical function given by the model of the measurement procedure. By the convention adopted for international intercomparisons, the uncertainty results are reported in terms of a combined standard uncertainty, rather than an “expanded uncertainty” which uses a “coverage factor *k*.” NIST standardization and calibration reports, otherwise, uniformly provide uncertainty statements for an expanded uncertainty with *k* = 2.

An uncertainty model for the CIEMAT/NIST tracing method has been developed by Collé and Zimmerman [18]. A related, but more detailed, uncertainty analysis for ^63^Ni assayed by the CIEMAT/NIST method (similar to that given in Table 16) has recently been given by Zimmerman and Collé [23].

Table 17 contains a summary of the analysis of the measurement uncertainty of the massic activity of the EUROMET ^55^Fe solution as obtained from direct comparative measurements against a NIST ^55^Fe standard. The uncertainty in the assay is dominated by the uncertainty of the NIST standard.

## 5. Intercomparison Findings

### 5.1 EUROMET ^63^Ni

**Fig. 4:**
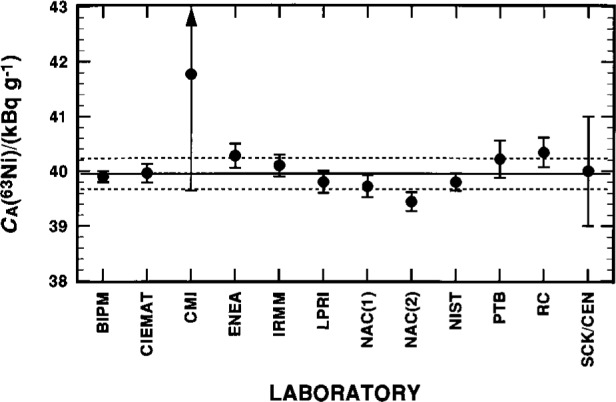
Results for the massic activity *C*_A_(^63^Ni) of the EUROMET ^63^Ni solution (in units of kBq · g^–1^) as reported by the various participating national metrological laboratories. The uncertainty bars on each datum correspond to a combined standard uncertainty as reported by the respective laboratory. The solid and broken lines represent, respectively, the mean *C*_A_(^63^NI) and the one standard deviation interval as obtained from averaging the results from 10 laboratories (excluding the values from CMI and SCK/CEN); see text.

The ^63^Ni assay results, as reported by eleven participating laboratories and as tabulated by Cassette [9], are briefly summarized in Table 18, and graphically displayed in Fig. 4. Cassette [9, 10] has also compiled additional details on each laboratory’s experimental aspects, such as the characteristics of their respective spectrometers, the scintillants employed, source preparation, counting conditions, quenching ranges, and estimated uncertainty components.

The unweighted mean of all 12 results (one laboratory, NAC, reported two values by different methods) is *C*_A_(^63^Ni) = 40.11 kBq · g^–1^ with a relative standard deviation of the mean of υ_m_ = 0.42 %.

Two of the laboratories, however did not use “absolute” (*sic*) methods, and their results are largely not considered in the discussion and analyses that follow. Both of these laboratories also had combined standard uncertainties that were well outside the range given by the other nine laboratories. The first of these (CMI) employed a method in which the activity was derived from extrapolating the coincidence counting rates as a function of the ratio of the coincident-to-sum count rates. Their value is clearly inhomogeneous with those reported by the other laboratories. The second laboratory (SCK/CEN) performed only comparative LS measurements against an LPRI-derived ^63^Ni standard. This result therefore is highly correlated to the LPRI value and can not be considered to be an independent value.

**Fig. 5:**
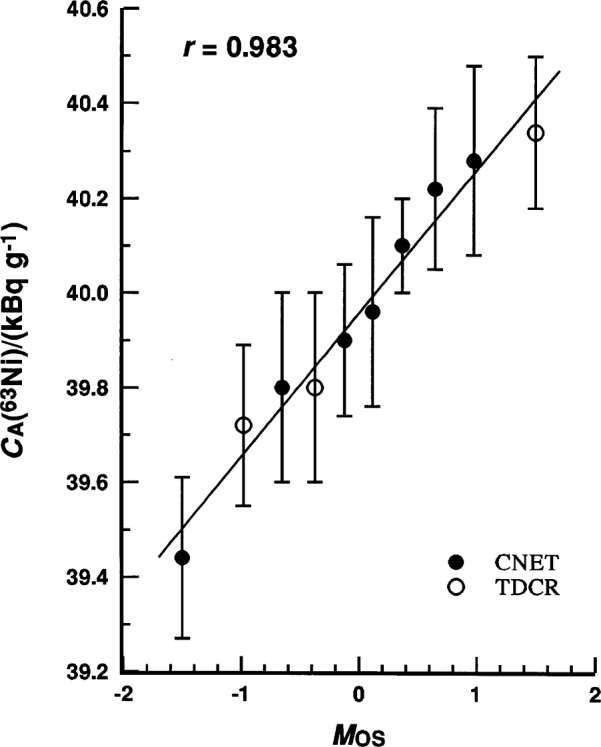
Normal probability plot for the reported massic activity *C*_A_(^63^Ni) of the EUROMET ^63^Ni solution (in units of kBq · g^–1^) as reported by 10 participating national metrological laboratories (see Fig. 4). The abscissa is the order statistic medians *M*_OS_ from a normal *N*(0,1) distribution as given by Filliben [52]. The test statistic *r* is the normal probability plot correlation coefficient.

The remaining 10 results have an unweighted mean of *C*_A_(^63^Ni) = 39.956 kBq · g^–1^ with *υ*_m_ = 0.22 %. The weighted mean and its relative weighted standard deviation, obtained with weighting factors of the reciprocals of the square of the reported combined standard uncertainties (given in Table 18), are statistically equivalent with *C*_A_(^63^Ni) = 39.891 kBq · g^–1^ and *υ*_wm_ = 0.14 %. The 10 measurement values are homogeneous, and may be considered to be normally distributed based on the normal probability plot given in Fig. 5 and its correlation coefficient *r*-test statistic. The normal probability plot correlation coefficient *r*, defined as the product moment correlation coefficient between the ordered observations and the order statistic medians *M*_os_ from a normal *N*(0,1) distribution [52], in this case has a value of *r* = 0.983. Based on the percent points *p* of *r* for a sample size of *n* = 10, as given by Filliben [52], the observed *r* lies between the *p* = 75 % and *p* = 90 % points of the null distribution, and is well above the *p* = 5 % critical value (where *p* may be interpreted to be the probability that the observations are not non-normally distributed). Hence, there is no evidence to support non-normality in the data (i.e., it does not contradict the *r*-test hypothesis of normality).

Of these 10 results, seven were based on use of the CIEMAT/NIST ^3^H-standard efficiency tracing (CNET) method and three were based on the triple-to-double coincidence ratio (TDCR) method. The unweighted mean *C*_A_(^63^Ni) and relative standard deviation of the mean *υ*_m_ obtained by the two methods are: *C*_A_(^63^Ni) = 39.957 kBq · g^–1^ with *υ*_m_ = 0.27 % (*n* = 7) for the CNET method; and *C*_A_(^63^Ni) = 39.953 kBq · g^–1^ with *υ*_m_ = 0.49 % (*n* = 3) for the TDCR method. Thus, there are no apparent, substantive method-dependent differences in the reported values. This conclusion, however, is not as straightforward as it might initially appear. It is somewhat clouded in that the TDCR results are, as noted by Cassette [9, 10], largely independent of the employed ^63^Ni *E_β_*_(max)_, whereas the CNET results, being computationally-based on calculated Fermi distributions for the ^63^Ni spectrum using the EFFY codes [31–33], are dependent on the chosen *E_β_*_(max)_. The cloudiness arises from the fact that all of the laboratories, except NIST, employed the intercomparison’s “recommended” nuclear data set (see Table 1), which is not the “best available” data [1, 18] and which has been acknowledged by Cassette [9, 10] to be “untenable” in lieu of more recent and critical data evaluations. Fortunately, the *C*_A_(^63^Ni) values reported by NIST are sufficient to sort out this convolution to a first approximation. If one were to blindly compare the reported NIST value *C*_A_(^63^Ni) = 39.80 kBq · g^–1^ to just the other laboratories using the CNET method, then one would obtain an evident nuclear-data-based “error” (in the true sense) because of the difference in the *E_β_*_(max)_ input assumptions. With the change in assumed *E_β_*_(max)_, as given in Sec. 4.1, the NIST value would increase by approximately 0.40 % to 0.45 % to give a revised value of *C*_A_(^63^Ni) = 40.0 kBq · g^–1^. This latter value is the only one which should be compared to any other laboratory using the CNET method. As a result, the initially apparent 0.4 % difference between the NIST *C*_A_(^63^Ni) value and the mean *C*_A_(^63^Ni) for the CNET results (i.e., 39.80/39.96 = 0.996 compared to 40.0/39.96 = 1.000) entirely disappears.

Using this same logic, it becomes apparent that the comparison between the two methods is indeed clouded due to nuclear-data dependencies. If one assumes that all of the results obtained with the CNET method should be decreased (due to the change in *E_β_*_(max)_) by about the same factor that was observed by NIST, then the initial excellent agreement between methods (39.95/39.96 = 1.000) would slightly worsen (39.95/39.8 = 1.004). The exact magnitude of this possible difference would depend on the efficiency ranges and experimental conditions employed by the other laboratories using the CNET method. In any case, this possible method difference, if it exists, is small. The magnitudes of the relative experimental standard deviations of the mean *υ*_m_ for the two methods preclude a definitive statement as to its existence. Its possible magnitude (circa 0.4 %), nevertheless, could rival the individual standard uncertainties reported by the laboratories (see Table 18).

Two other aspects of the results from the laboratories that used the CNET method are of considerable interest. Firstly, as noted in Table 18, the very good measurement agreement amongst the laboratories was obtained using ^3^H standards for the efficiency tracing that had considerably different origins (from six laboratories). This was a very encouraging finding. Zimmerman and Collé [53] recently made a more direct comparison between the LPRI and NIST ^3^H standards used in this work and found very good agreement between standards. Secondly, the EFFY or EFFY-equivalent codes used by the various laboratories were also very different (see Table 18). This too was a significant and encouraging finding. These codes are known to exhibit substantive differences in the calculated efficiencies ϵ for a given figure of merit *M* (see Sec. 2.2). For example, comparisons of the EFFY5 version used by CIEMAT [54] to the EFFY4 version used by NIST (this work) or to the earlier EFFY2 version used by NIST [16, 33] all have different *ϵ* (^3^H)/*ϵ* (^63^Ni) ratios for a given *M*. Despite these obvious underlying differences in the assumed *M* definitions, all three codes result in virtually identical tracing results for ^63^Ni. This was verified by NIST, as part of this work, by performing the 3H-standard efficiency tracing for two select sets of data with the three codes. Hence, it seems clear that the efficiency tracing can be performed adequately, irrespective of the choice of available codes, provided that only one code is consistently employed. In contrast, for example, if one code is used for the *ϵ* (^3^H) versus *M* relation and another code for the *M* versus *ϵ* (^63^Ni) relation, then the “error” (in the true sense) in the efficiency tracing might be significant, and actually dominate the overall measurement uncertainty. These observations also support our recent contentions that one must not try to place too much emphasis on the physical meaning of the figure of merit *M* [24, 35].

### 5.2 EUROMET ^55^Fe

**Fig. 6:**
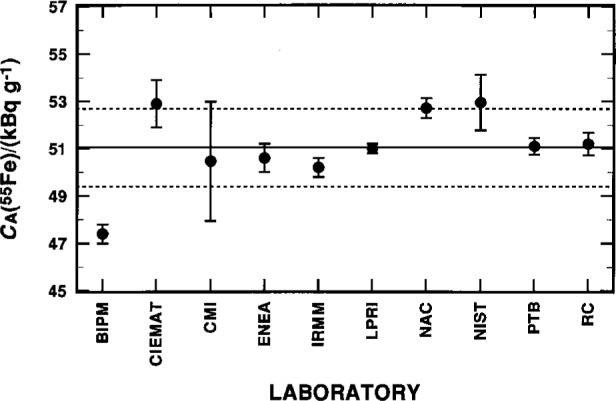
Results for the massic activity *C*_A_(^55^Fe) of the EUROMET ^55^Fe solution (in units of kBq · g^–1^) as reported by the various participating national metrological laboratories. The uncertainty bars on each datum correspond to a combined standard uncertainty as reported by the respective laboratory. The solid and broken lines represent, respectively, the mean *C*_A_(^55^Fe) and the one standard deviation interval as obtained from averaging the results from the 10 laboratories.

The “final” *C*_A_(^55^Fe) results reported by ten laboratories are given in Table 19 and Fig. 6. These values constitute a smaller subset of 17 independent determinations, which arose from some of the laboratories (e.g., IRMM, LPRI, and NAC) having performed measurements by several measurement methods. A more complete compilation of these results has been given by Cassette [9, 10]. The values given in Table 19 and Fig. 6 are each laboratory’s assessment of their “best estimate” for *C*_A_(^55^Fe).

The unweighted mean *C*_A_(^55^Fe) and its *υ*_m_ are *C*_A_(^55^Fe) = 50.05 kBq · g^–1^ and *υ*_m_ = 1.0 % (*n* = 10). The weighted mean and its *υ*_wm_ (weighted as before) are *C*_A_(^55^Fe) = 50.77 and *υ*_wm_ = 0.25 %.

**Fig. 7:**
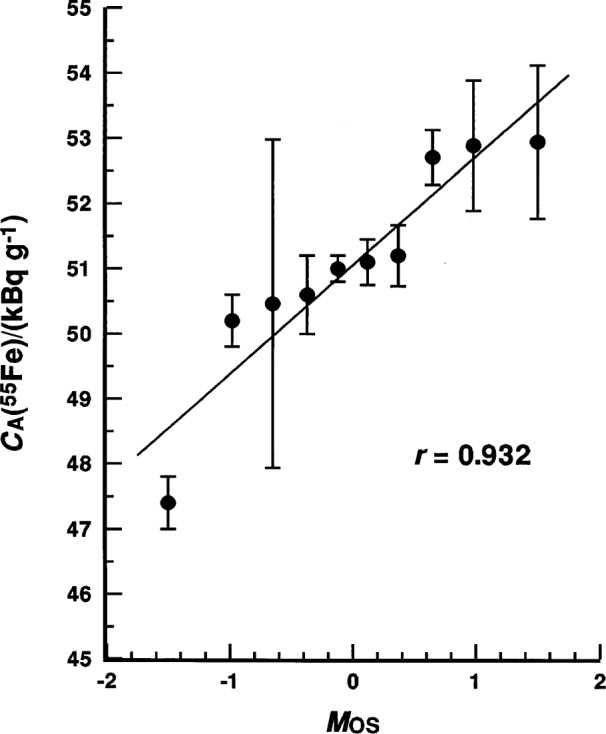
Normal probability plot for the reported massic activity *C*_A_(^55^Fe) of the EUROMET ^55^Fe solution (in units of kBq · g^–1^) as reported by 10 participating national metrological laboratories (see Fig. 6). The abscissa is the order statistic medians *M*_OS_ from a normal *N*(0,1) distribution as given by Filliben [52]. The test statistic *r* is the normal probability plot correlation coefficient.

The dispersion of the reported *C*_A_(^55^Fe) values may be somewhat discouraging (particularly to the responsible metrologists of the various national laboratories), but the values do not necessarily constitute an inhomogeneous data set. As shown in Fig. 7, the reported *C*_A_(^55^Fe) values may be considered to be normally distributed based on the *r*-test statistic. Alas, they just seem to be sampled from a distribution having a relatively large variance. The *r* = 0.932 for this case is well above the *p* = 5 % critical value, and falls just below *p* = 70 %. Nevertheless, the findings are disconcerting and seem to have structure in that the reported values appear to occur in three clusters: (i) a single low-lying “outlier” (BIPM); (ii) a high-lying group of three (CIEMAT, NIST, and NAC); and (iii) a six-laboratory majority at midrange. This structure in the values is apparent in both Figs. 6 and 7. Cassette [9, 10] established that there were no readily evident correlations between the reported values (or the aforementioned structure) and the employed measurement methods, or that the data was insufficient to establish consistency with any one particular method. He further concluded that the quality of the data did not warrant attempts at further statistical analyses of the reported values to extract an “average” internationally-accepted consensus value for the ^55^Fe massic activity *C*_A_(^55^Fe) of the EUROMET solution.

The findings are also disconcerting in that the observed differences amongst the laboratories are considerably greater than that desired by the various national metrological laboratories. In fact, these findings are only marginally better than those obtained from an international ^55^Fe measurement intercomparison that was conducted nearly 20 years earlier (see Sec. 1.6).

Cassette [9, 10] has adequately addressed many of the attendant problems with the assay of ^55^Fe by LS spectrometric methods. The incompatibility of efficiency tracing codes for those using (or attempting to use) the CNET method was observed not only by NIST (Sec. 4.3), but was also noted by ENEA and PTB. This may also be a factor in the results of BIPM and NAC. Many laboratories similarly reported on various observed and inexplicable cocktail composition effects. Measurement differences were observed from use of different commercially prepared scintillants, and/or from the quantity and composition of what was added to the scintillants. These discrepancies are now not necessarily surprising in light of our recent investigations into cocktail composition effects in low-energy LS spectrometry [27–29].

## 6. Concluding Comments

This compendium largely documents all aspects of the results obtained by NIST for the present international measurement intercomparison of ^63^Ni and ^55^Fe by LS spectrometric methods.

These NIST results (*vis à vis* with those of the other national radionuclidic metrology laboratories) can be used to highlight several salient points:

1. The international compatibility of standardizations of low-energy *β* emitters, like ^63^Ni, appears to be well in hand using either the CNET or TDCR methodologies. Whether the methods can be comparably applied to any other low-energy *β*-emitting radionuclide may again depend upon having an understanding of the cocktail composition effects for that case.
2. The NIST standardization of ^63^Ni is in excellent agreement with this international community’s consensus value for the ^63^Ni massic activity of the distributed EUROMET solution. Based on concurrent measurements performed for this work, the recent standardization [23] of a ^63^Ni solution standard (SRM 4426C) was verified, and its certified value is in excellent agreement with the ^63^Ni standardizations performed by the other national metrology laboratories that participated in this EUROMET intercomparison.
3. It would be useful if the assumed absence of the slight possible difference (*circa* 0.4 %) between the two methods, that may or may not exist, was independently verified by additional inter-laboratory measurements that would remove the present nuclear-data-based *E_β_*_(max)_ bias assumptions.
4. The standardizations of low-*Z* nuclides that decay by electron capture (EC), like ^55^Fe, are as problematic as ever. The dispersion of the results from the national metrology laboratories is such that it is impossible to arrive at an internationally accepted consensus value for the ^55^Fe massic activity of the distributed EUROMET solution.
5. Use of the CNET method for such EC-nuclide standardizations clearly requires a greater compatibility between the existing software codes if one is going to use ^3^H standards for efficiency tracing.
6. The inter-laboratory discrepancies observed for ^55^Fe may not solely have “origins” in the “measurement methods used,” as has been suggested by Cassette [9], but may also have a large component from poorly understood cocktail composition effects [27–29].
7. The NIST standardization of ^55^Fe has an unacceptably large (approximately 4 %) difference from a majority of the other national metrological laboratories. This point prompts us to not only critically evaluate our measurement capability for low-*Z*, EC-decaying nuclides, but also to continue our collaborations with our sister national laboratories in improving such standardizations.

## Figures and Tables

**Table 1:** Principal nuclear data for ^3^H and ^63^Ni decay as “recommended” by EUROMET for use in the intercomparison and those used by NIST. Each quoted uncertainty is a standard uncertainty (an assumed standard deviation).

Parameter	Value “recommended” by EUROMET		Value used by NIST	
Radionuclide	^3^H	^63^Ni	^3^H	^63^Ni
*T*/a	12.34 ± 0.02	100.1 ± 2.0	12.34 ± 0.02^a^	101.1 ± 1.4
*E_β_*_(max)_/keV	18.619 ± 0.011	65.87 ± 0.20	18.594 ± 0.008	66.945 ± 0.004
*E_β_*_(man)_/keV	5.71 ± 0.03	17.13 ± 0.05	5.69 ± 0.04^b^	17.426 ± 0.013^b^
*β* transition	allowed	allowed	allowed	allowed
*β* spectrum shape factor	1	1	1	1

a This half-life was used for all ^3^H decay corrections in this intercomparison, i.e., for the time intervals from the reference times of the employed ^3^H standards (Table 5) to the measurement times. A half-life of 12.33 a ± 0.06 a was used for prior decay corrections to the reference time for the NIST ^3^H standard.

b The values of *E_β_*_(mean)_ are not required as input into the EFFY code, but rather are calculated by EFFY from input values of *E_β_*_(max)_. The *E_β_*_(mean)_ values tabulated here are those obtained from independent evaluations. For comparison, those obtained from the EFFY4 code (using the tabulated *E_β_*_(max)_ values) are *E_β_*_(mean_ = 5.71 keV for ^3^H and *E_β_*_(mean)_ = 17.43 keV for ^63^Ni.

**Table 2:** Principal nuclear and atomic data for ^55^Fe decay as “recommended” by EUROMET for use in the intercomparison and as used by NIST. Each quoted uncertainty is a standard uncertainty (an assumed standard deviation)

Parameter	Value “recommended” by EUROMET	Value used by NIST
Half-life	2.735 a ± 0.022 a	2.735 a ± 0.022 a
Total EC probability to ground state in ^55^Mn (231.6 keV transition)	1	1
K-capture probability	0.881 ± 0.004	0.881
L-capture probability	0.103 ± 0.004	0.103
(M + N)-capture probability	0.0161 ± 0.0008	0.016
K-shell fluorescence yield	0.321 ± 0.007	0.32
Average L-shell fluorescence yield	0.0053 ± 0.0004	0.0030
Average energy Kα x ray (and *P*_Kα_ probability)	5.895 keV	5.89 keV (0.841)
Average energy K*β* x ray (and *P*_K_*_β_* probability)	6.505 keV	6.52 keV (0.149)
Average energy Lα x ray	0.635 keV	0.63 keV
Average energy K-LL Auger electron (and *P*_K-LL_ probability)	5.08 keV	5.08 keV (0.8024)
Average energy K-LM Auger electron (and *P*_K-LM_ probability)	5.80 keV	5.78 keV (0.1822)
Average energy K-MM Auger electron (and *P*_K-MM_ probability)	6.45 keV	6.42 keV (0.0154)
Average energy L-MM Auger electron	0.62 keV	0.65 keV

**Table 3:** Characteristics of the NIST LS spectrometers employed for the EUROMET intercomparison

Characteristic	System B	System P
LS spectrometer model	Beckman LS7800	Packard Tri-carb A2500TR
Operating mode	sum-coincidence	sum-coincidence
Photomultiplier tubes	Hamamatsu R331-05	Hamamatsu R331-08
Operating temperature	ambient	ambient
Coincidence resolving time	22 ns	18 ns
Sum-coincident pulse amplification	logarithmic	linear
Pulse resolving time	5 *μ*s to 33 *μ*s (variable with pulse height)	12 *μ*s (fixed)
Spectral analog-to-digital converter (ADC) capacity	1000 channels	2048 channels
Nominal conversion gain energy per channel)	variable (with logarithmic energy)	≅ 1 keV
Detection threshold (nominal)	≤ 1 keV	≤ 1 keV
Live-time determination method (and standard uncertainty)	gated oscillator (scaled) (± 0.1 %)	gated oscillator (scaled) (± 0.1 %)
Quench indicating parameter (QIP)	Horrocks number (H)	transformed Spectral Index of the External Standard (tSIE) (proprietary)
External *γ*-ray source for QIP determination (and location)	^137^Cs (side)	^133^Ba (bottom)

**Table 4:** Scintillants (commercially-prepared) used by NIST for the EUROMET intercomparison

Commercial scintillant	Acronym descriptor	Manufacturer	Density (g · mL^–1^)	Composition
Ready Safe	RS	Beckman	0.97	phenylxylylethane (PXE) 50 % to 80 %; non-ionic surfactant 20 % to 50 %; 2,5-diphenyl-oxazole (PPO) < 1 %
Ultima-Gold and Ultima-Gold AB	UG	Packard	0.96	di-isopropylnapthalene (DIN); with emulsifiers; PPO and bis(2-methylstyryl)benzene (bis-MSB)
Instagel XF	IG	Packard	≅ 0.9	1,2,4-trimethylbenzene(pseudocumene); with emulsifiers; PPO and bis-MSB
PCS	PCS	Amersham	0.92	xylene; 2-ethoxyethanol; unspecified fluor

**Table 5:** Summary of the radionuclidic solutions employed by NIST for the EUROMET intercomparison

Radionuclidic solution	Solution composition	Massic activity (kBq · g^–1^)	Reference time
NIST ^3^H^b^	tritiated H_2_O	69.23 ± 0.34	1700 UT 15 Aug. 1995
LPRI ^3^H^b^	tritiated H_2_O	209.8 ± 1.1	1200 UT 27 Jan. 1994
NIST ^63^Ni^c^	1 mol · L^–1^ HCl 98 *μ*g · g^–1^ Ni^+2^ (1.016 ± 0.002) g · mL^–1^	50.53 ± 0.23	1700 UT 15 Aug. 1995
EUROMET ^63^Ni	1 mol · L^–1^ HCl 36 *μ*g · g^–1^ Ni^+2^	≅ 40	1200 UT 1 Jan. 1996
NIST ^55^Fe^d^	1 mol · L^–1^ HCl 21.3 *μ*g · g^–1^ Fe^+3^ (1.015 ± 0.001) g · mL^–1^	22.10 ± 0.46	1200 UT 1 Jan. 1996
EUROMET ^55^Fe	1 mol · L^–1^ HCl 2.8 *μ*g · g^–1^ Fe^+3^	≅ 50	1200 UT 1 Jan. 1996

a The quoted uncertainties of the massic activity are standard uncertainties.

b Based on a gravimetric dilution of NIST SRM 4927E, and decay over 16.95 a using a ^3^H half-life of *T* = (12.33 ± 0.06) a. The relative standard uncertainty in the ^3^H primary calibration (in 1978 by internal gas-proportional counting) was 0.18 %.

c LPRI, Calibration Certificate 94/R-007E.

d NIST SRM 4226C (1995).

e NIST SRM 4929E (1996).

**Table 6:** LS cocktail compositions used by NIST for the EUROMET intercomparison of ^63^Ni

Cocktail series	Cocktail identity	Number of samples	Radionuclidic solution^a^	Scintillant^b^	Cocktail composition^c^	Cocktail component parameters^d^
A	F1 – F7	7	EUROMET ^63^Ni	UG	10.33 g UG 0.7 g H_2_O 0.009 mol EDTA^–2^ 41 mg aliquant	*m* = 11.2 *f*_w_ = 0.0745 *c*_HCl_ = 0.049 *m*_Ni_ = 1.46
A	N1 – N7	7	NIST ^63^Ni	UG	10.33 g UG 0.7 g H_2_O 0.009 mol EDTA^–2^ 40 mg aliquant	*m* = 11.2 *f*_w_ = 0.0745 *c*_HCl_ = 0.048 *m*_Ni_ = 3.88
A	TF1 – TF7	7	LPRI ^3^H	UG	10.33 g UG 0.7 g H_2_O 0.009 mol EDTA^–2^ 15 mg Ni^+2^ carrier 15 mg aliquant	*m* = 11.2 *f*_w_ = 0.0737 *c*_HCl_ = 0.019 *m*_N_ = 1.53
A	TN1 – TN7	7	NIST ^3^H	UG	10.33 g UG 0.7 g H_2_O 0.009 mol EDTA^–2^ 30 mg Ni^+2^ carrier 38 mg aliquant	*m* = 11.2 *f*_w_ = 0.0769 *c*_HCl_ = 0.037 *m*_Ni_ = 3.07
B	F8 – F14	7	EUROMET ^63^Ni	UG	10.33 g UG 0.6 g H_2_O 66 mg aliquant	*m* = 10.9 *f*_w_ = 0.056 *c*_HCl_ = 0.10 *m*_Ni_ = 4.0
B	N8 – N14	7	NIST ^63^Ni	UG	10.33 g UG 0.6 g H_2_O 65 mg aliquant	*m* = 10.9 *f*_w_ = 0.056 *c*_HCl_ = 0.06 *m*_Ni_ = 6.0
B	TF8 – TF14	7	LPRI ^3^H	UG	10.33 g UG 0.6 g H_2_O 15 mg Ni^+2^ carrier 23 mg aliquant	*m* = 10.9 *f*_w_ = 0.056 *c*_HCl_ = 0.03 *m*_Ni_ = 2.45
B	TN8 – TN14	7	NIST ^3^H	UG	10.33 g UG 0.6 g H_2_O 30 mg Ni^+2^ carrier 65 mg aliquant	*m* = 10.9 *f*_w_ = 0.056 *c*_HCl_ = 0.05 *m*_Ni_ = 4.9
C C	F15 – F21 N15 – N21	7 7	EUROMET ^63^Ni NIST ^63^Ni	UG UG	10.24 g UG 17 mg H_2_O 28 mg aliquant 10.24 g UG 17 mg H_2_O 32 mg aliquant	*m* = 10.3 *f*_w_ = 0.0044 *c*_HCl_ = 0.62 *m*_Ni_ = 1.01 *m* = 10.3 *f*_w_ = 0.0048 *c*_HCl_ = 0.65 *m*_Ni_ = 3.14
C C	TF15 –TF21 TN15 – TN21	7 7	LPRI 3H NIST ^3^H	UG UG	10.24 g UG 14 mg Ni^+2^ carrier 17 mg aliquant 10.24 g UG 28 mg Ni^+2^ carrier 50 mg aliquant	*m* = 10.3 *f*_w_ = 0.0030 *c*_HCl_ = 0.45 *m*_Ni_ = 1.67 *m* = 10.3 *f*_w_ = 0.0076 *c*_HCl_ = 0.36 *m*_Ni_ = 2.74
D	F22 – F28	7	EUROMET ^63^Ni	PCS	10.28 PCS 36 mg aliquant	*m* = 10.3 *f*_w_ = 0.0035 *c*_HCl_ = 1 *m*_Ni_ = 1.30
D D	N22 – N28 TN22 – TN 28	7 7	NIST ^63^Ni NIST ^3^H	PCS PCS	10.28 g PCS 32 mg aliquant 10.28 mg PCS 21 mg Ni^+2^ carrier 19 mg aliquant	*m* = 10.3 *f*_w_ = 0.0031 *c*_HCl_ = 1 *m*_Ni_ = 3.14 *m* = 10.3 *f*_w_ = 0.0039 *c*_HCl_ = 1 *m*_Ni_ = 2.06
E E	F29 – F35 N29 – N35	7 7	EUROMET ^63^Ni NIST ^63^Ni	PCS PCS	9.80 g PCS 0.6 g H_2_O 36 mg aliquant 9.80 g PCS 0.6 g H_2_O 32 mg aliquant	*m* = 10.4 *f*_w_ = 0.0609 *c*_HCl_ = 0.057 *m*_Ni_ = 1.30 *m* = 10.4 *f*_w_ = 0.0606 *c*_HCl_ = 0.051 *m*_Ni_ = 3.14
E	TN29 – TN35	7	NIST ^3^H	PCS	9.80 g PCS 0.6 g H_2_O 21 mg Ni^+2^ carrier 19 mg aliquant	*m* = 10.4 *f*_w_ = 0.0613 *c*_HCl_ = 0.033 *m*_Ni_ = 2.06

a Refer to Table 5.

b Refer to Table 4.

c Exclusive of a variable quantity of the imposed chemical quenching agent (refer to text).

d *m* = total mass (in grams) of cocktail; *f*_w_ = H_2_O mass fraction in cocktail; *c*_HCl_ = HCl concentration (mol · L^–1^) in aqueous fraction of cocktail; *m* = total mass of Ni^+2^ Ni (in *μ*g) in cocktail.

**Table 7:** LS cocktail compositions used by NIST for the EUROMET intercomparison of ^55^Fe

Cocktail series	Cocktail identity	Number of samples	Radionuclidic solution^a^	Scintillant^b^	Cocktail composition^c^	Cocktail component parameters^d^
F	F1 – F7	7	EUROMET ^55^Fe	UG	9.91 g UG 39 mg aliquant	*m* = 9.95 *f*_w_ = 0.004 *c*_HCl_ = 1 *m*_Fe_ = 0.11
F	N1 – N7	7	NIST ^55^Fe	UG	9.91 g UG 44 mg aliquant	*m* = 9.95 *f*_w_ = 0.004 *c*_HCl_ = 1 *m*_Fe_ = 0.93
G	FW1 – FW7	7	EUROMET ^55^Fe	UG	9.51 g UG 0.6 g H_2_O 33 mg aliquant	*m* = 10.2 *f*_w_ = 0.062 *c*_HCl_ = 0.05 *m*_Fe_ = 0.09
G	NW1 – NW7	7	NIST ^55^Fe	UG	9.51 g UG 0.6 g H_2_O 42 mg aliquant	*m* = 10.2 *f*_w_ = 0.063 *c*_HCl_ = 0.065 *m*_Fe_ = 0.89
G	XW1 – XW7	7	EUROMET *and* NIST ^55^Fe	UG	9.51 g UG 0.6 g H_2_O 40 mg aliquants both	*m* = 10.2 *f*_w_ = 0.067 *c*_HCl_ = 0.12 *m*_Fe_ = 0.96
G	TW1 – TW7	7	NIST 3H	UG	9.51 g UG 0.6 g H_2_O 40 mg HCl soln. 39 mg aliqaunt	*m* = 10.2 *f*_w_ = 0.067 *c*_HCl_ = 0.12 *m*_Fe_ = 0
H	FA1 – FA6	6	EUROMET ^55^Fe (adjusted)	UG	9.36 g UG 0.6 g H_2_O 55 mg aliquant	*m* = 10.0 *f*_w_ = 0.065 *c*_HCl_ = 0.084 *m*_Fe_ = 0.98
H	NA1 – NA6	6	NIST ^55^Fe	UG	9.36 g UG 0.6 g H_2_O 51 mg aliquant	*m* = 10.0 *f*_w_ = 0.069 *c*_HCl_ = 0.078 *m*_Fe_ = 1.09
I	FY1 – FY4	3	EUROMET ^55^Fe	UG	9.36 g UG 32 mg aliquant 0 to 55 mg carrier soln.	*m* = 9.4 *f*_w_ = 0.003 to 0.009 *c*_HCl_ = 1 *m*_Fe_ = 0.095 to 0.64
I	NY1 – NY4	4	NIST ^55^Fe	UG	9.36 g UG 17 to 32 mg aliquant 0 to 37 mg carrier soln.	*m* = 9.4 *f*_w_ = 0.002 to 0.007 *c*_HCl_ = 1 *m*_Fe_ = 0.35 to 1.05

a Refer to Table 5.

b Refer to Table 4.

c Exclusive of a variable quantity of the imposed chemical quenching agent (refer to text).

d *m* = total mass (in grams) of cocktail; *f*_w_ = H_2_O mass fraction in cocktail; *c*_HCl_ = HCl concentration (mol · L^–1^) in aqueous fraction of cocktail; *m*_Fe_ = total mass of Fe^+3^ (in *μ*g) in cocktail.

**Table 8:** NIST experiments (CIEMAT/NIST efficiency tracing) for the EUROMET intercomparison of ^63^Ni

Experiment number	Cocktail series	Spectrometer	^3^H efficiency range	QIP range	Cocktail age (d)	Experimental objective	Comments^a^
1	A	B	0.43–0.38	*H* = 68–94	< 1–3	Trace EUROMET ^63^Ni and NIST ^63^Ni against LPRI ^3^H and NIST ^3^H CIEMAT/NIST	High *f*_w_; reasonably matched cocktails; chelated
2	A	P	0.48–0.45	*tSIE* = 523–420	3 to 6	Trace EUROMET ^63^Ni and NIST ^63^Ni against LPRI ^3^H and NIST ^3^H CIEMAT/NIST	High *f*_w_; reasonably matched cocktails; chelated
3	B	B	0.44–0.39	*H* = 63–89	< 1 to 4	Trace EUROMET ^63^Ni and NIST ^63^Ni against LPRI ^3^H and NIST ^3^H CIEMAT/NIST	High *f*_w_; reasonably matched cocktails; not chelated
3^b^	B	B		*H* = 65–89	< 1 to 4	Trace EUROMET ^63^Ni against NIST ^63^Ni CIEMAT/NIST	High *f*_w_; see above excpt. 3
4	B	P	0.50–0.46	*tSIE* = 541–433	< 1 to 4	Trace EUROMET ^63^Ni and NIST ^63^Ni against LPRI ^3^H and NIST ^3^H CIEMAT/NIST	High *f*_w_; reasonably matched cocktails; not chelated
5	C	B	0.50–0.42	*H* = 36–79	< 1 to 3	Trace EUROMET ^63^Ni and NIST ^63^Ni against LPRI ^3^H and NIST ^3^H CIEMAT/NIST	Results not used; low *f*_w_;
6	C	P	0.56–0.48	*tSIE* = 673–465	4 to 7	Trace EUROMET ^63^Ni and NIST ^63^Ni against LPRI ^3^H and NIST ^3^H CIEMAT/NIST	Results not used; low *f*_w_;
7	B	B	0.44–0.38	*H* = 62–88	15 to 18	Trace EUROMET ^63^Ni and NIST ^63^Ni against LPRI ^3^H and NIST ^3^H CIEMAT/NIST	High *f*_w_; aged cocktail
8	D	B	0.47–0.36	*H* = 63–114	1 to 3	Trace EUROMET ^63^Ni and NIST ^63^Ni against only NIST ^3^H CIEMAT/NIST	Results not used; low *f*_w_; alternate (xylene) cocktails
9	E	B	0.41–0.31	*H* = 87–139	< 1 to 3	Trace EUROMET ^63^Ni and NIST ^63^Ni against *only* and NIST ^3^H CIEMAT/NIST	High *f*_w_; alternate; (xylene) cocktails

a *f*_w_ = H_2_O mass fraction in cocktail.

**Table 9:** NIST experiments for the EUROMET intercomparison of ^55^Fe

Experiment number	Cocktail series	Spectrometer	^55^Fe efficiency range	QIP range	Experimental Objective	Comments^a^
10	F	B	0.49–0.35	*H* = 39–88	Compare EUROMET ^55^Fe against NIST ^55^Fe (with quench corrections)	Results not used; low *f*_w_; traced with *m*_Fe_ = 0.11 against 0.93 cocktails
11	G	B	0.43–0.28	*H* = 64–112	Compare EUROMET ^55^Fe against NIST ^55^Fe (with quench corrections)	High *f*_w_; ratio *m*_Fe_ = 0.09/0.89
12	G	B	0.43–0.28	*H* = 64–112	Trace EUROMET ^55^Fe against NIST ^55^Fe using EMI code	High *f*_w_; ratio *m*_Fe_ = 0.09/0.89
13	G	B	0.43–0.28	*H* = 64–112	Trace EUROMET ^55^Fe against NIST ^3^H using EMI and EFFY4 codes	Results not used; high *f*_w_; ratio *m*_Fe_ = 0.09/0
14	G	B	0.43–0.28	*H* = 64–112	Compare EUROMET ^55^Fe against NIST ^55^Fe by standard additions	High *f*_w_; ratio *m*_Fe_ = 0.98/0.89
15	G	P	0.52–0.36	*tSIE* = 544–357	Compare EUROMET ^55^Fe against NIST ^55^Fe (with quench corrections)	High *f*_w_; ratio *m*_Fe_ = 0.090/0.89 aged (10 d) cocktails; alternate spectrometer
16	H	B	0.42–0.29	*H* = 69–113	Compare EUROMET ^55^Fe against NIST ^55^Fe (with quench corrections) after adjust Fe^+3^	High *f*_w_; ratio *m*_Fe_ = 0.98/1.09;
17	I	B	0.49 –0.45	*H* = 36–44	Compare EUROMET ^55^Fe against NIST ^55^Fe for varying cocktail compositions	Results not used; low *f*_w_; ratio *m*_Fe_ variable

a *f*_w_ = H_2_O mass fraction in cocktail; *m*_Fe_ = total mass of Fe^+3^ (in *μ*g) in cocktail.

**Table 10:** Results for the massic activity *C*_A_(^63^Ni) of the EUROMET ^63^Ni solution (in units of Bq · g^–1^) as obtained by NIST from efficiency tracing against a LPRI ^3^H standard and a NIST ^3^H standard using the CIEMAT/NIST methodology (employing the EFFY4 code)

Experiment number	*C*_A_(^63^Ni)/(Bq · g^–1^)^a^		
	Traced against LPRI ^3^H standard	Traced against NIST ^3^H standard	Traced against NIST ^63^Ni standard^b^
1	39775 ± 25	39775 ± 32	(39753)
2	39771 ± 53	39738 ± 58	(39709)
3	39926 ± 33	39809 ± 38	39902 ± 149
4	39863 ± 30	39756 ± 30	(39922)
7	39837 ± 51	39721 ± 50	(39754)
9	—	39605 ± 89	(39453)
Mean	39834 ± 65	39734 ± 70	39749 ± 169

Relative standard deviation	0.073 %	0.072 %	0.17 %

a The reference time for the massic activity *C*_A_(^63^Ni) is 1200 UT 1 January 1996. The uncertainties are one standard deviation estimates with *υ* = 6 degrees of freedom.

b The result for experiment 3 was derived explicitly by tracing the EUROMET ^63^Ni against the NIST ^63^Ni standard through the four F_1_, F_2_, F_3_, and F_4_ functions (see Sec. 2.2). The other values given here (in parentheses) were inferred from the tracing results given in Table 12.

**Table 11:** Results (for comparison only) for the massic activity *C*_A_(^63^Ni) of the EUROMET ^63^Ni solution (in units of Bq · g^–1^) as obtained by NIST for cocktails containing low *f*_w_ and which were considered (*a priori*) to result in unreliable tracing results (contrast with Table 10)

	*C* (^63^ A Ni)/(Bq · g^–1^)^a^	
Experiment number	Traced against LPRI ^3^H standard	Traced against NIST ^3^H standard
5	39080 ± 55	39072 ± 58
6	39092 ± 48	39068 ± 41
8		39462 ± 156

a The reference time for the massic activity *C*_A_(^63^Ni) is 1200 UT 1 January 1996. The uncertainties are one standard deviation estimates with *υ* = 6 degrees of freedom.

**Table 12:** Results for the massic activity *C*_A_(^63^Ni) of the NIST ^63^Ni standard (in units of Bq · g^–1^) as obtained by a NIST re-assay from efficiency tracing against a LPRI ^3^H standard and a NIST ^3^H standard using the CIEMAT/NIST methodology (employing the EFFY4 code)

	*C*_A_(^63^Ni)/(Bq · g^–1^)^a^	
Experiment number	Traced against LPRI ^3^H standard	Traced against NIST ^3^H standard
1	50367 ± 55	50376 ± 38
2	50352 ± 49	50315 ± 50
3	50577 ± 165	50432 ± 184
4	50507 ± 176	50372 ± 190
7	50437 ± 184	50300 ± 220
9		50207 ± 107

Mean	50448 ± 95	50334 ± 78
Relative standard deviation of the mean	0.084 %	0.063 %
Relative difference from certified value	+0.095 %	−0.13 %

a The reference time for the massic activity *C*_A_(^63^Ni) is 1200 UT 1 January 1996. The uncertainties are one standard deviation estimates with *υ* = 6 degrees of freedom.

**Table 13:** Results (for comparison only) for the massic activity *C*_A_(^63^Ni) of the NIST ^63^Ni solution (in units of Bq · g^–1^) as obtained by a NIST re-assay for cocktails containing low *f*_w_ and which were considered (*a priori*) to result in unreliable tracing results (contrast with Table 12)

	*C*_A_(^63^Ni)/(Bq · g^–1^)^a^	
Experiment number	Traced against LPRI ^3^H standard	Traced against NIST ^3^H standard
5	49773 ± 46	49762 ± 54
6	49720 ± 41	49689 ± 35
8		49776 ± 74

a The reference time for the massic activity *C*_A_(^63^Ni) is 1200 UT 1 January 1996. The uncertainties are one standard deviation estimates with *ν* = 6 degrees of freedom.

**Table 14:** Results for the massic activity *C*_A_(^55^Fe) of the EUROMET ^55^Fe solution (in units of Bq · g^–1^) as obtained by NIST from comparative measurements against a NIST ^55^Fe standard

Experiment number	*C* (^55^ A Fe)/Bq · g^–1^)^a^
11	52944 ± 420
12	52952 ± 447
14	52071 ± 1441
15	53443 ± 517
16	52956 ± 325

Unweighted mean	52873 ± 497
Relative standard deviation of the mean (unweighted)	0.42 6 %
Weighted mean	53010 ± 497
Relative standard deviation of the mean (weighted)	0.38 %

a The reference time for the massic activity *C*_A_(^55^Fe) is 1200 UT 1 January 1996. The uncertainties are one standard deviation estimates with *y* = 6 degrees of freedom (except *υ* = 5 for experiment 16).

**Table 15:** Results (for comparison only) for the massic activity *C*_A_(^55^Fe) of the EUROMET ^55^Fe solution (in units of Bq · g^–1^) as obtained by NIST for cocktails containing low *f*_w_ and which were considered (*a priori*) to result in unreliable results (contrast with Table 14)

Experiment number	*C*_A_(^55^Fe)/(Bq · g^–1^)^a^
10	52352 ± 300
13	46658 ± 383
17	51961 ± 522

a The reference time for the massic activity *C*_A_(^55^Fe) is 1200 UT 1 January 1996. The uncertainties are one standard deviation estimates with *υ* = 6 degrees of freedom (except *υ* = 2 for experiment 17).

**Table 16:** NIST uncertainty analysis for the EUROMET intercomparison of ^63^Ni. The analysis makes no estimate for any uncertainty due to the possible presence of radionuclidic impurities, nor for any uncertainty associated with the assumed model assumptions in the CIEMAT/NIST method

Item	Uncertainty component (and Type)	Relative uncertainty contribution to massic activity of ^63^Ni (%)
1	LS measurement variability; reproducibility with 7 cocktails of comparable composition; *υ* = 6 degrees of freedom (A)	0.034
2	LS sample variability (quench dependence); reproducibility between sample compositions; *υ* = 6 (A)	0.085
3	LS cocktail stability and composition effects; *υ* = 3 (A)	0.06
4	Gravimetric (mass) determinations for LS samples (B)	0.05
5	Experimental ^3^H efficiency from NIST ^3^H standard (B)	0.29
6	Background measurement variability; wholly embodied in item 1 above (A)	—
7	Spectrometer and scintillant dependencies; wholly embodied in items 1, 2, and 3 above (A)	—
8	Livetime determinations for LS counting time intervals; includes uncorrected deadtime effects (B)	0.07
9	Decay corrections for ^63^Ni and ^3^H (B)	0.001
10	Variability in determination of QIPs for ^3^H and ^63^Ni (A)	0.13
11	Precision of ^3^H efficiency versus figure of merit (*M*) calculations (step sizes) (B)	0.008
12	Fit of relation between ^3^H QIP and calculated *M* (B)	0.02
13	Precision of ^63^Ni efficiency versus figure of merit (M) calculations (step sizes) (B)	0.002
14	Fit of relation between calculated *M* and ^63^Ni efficiency (A)	0.002
15	Effect of ionization quenching assumptions on efficiency calculations (B)	0.1
16	Effect of asymmetry in phototube responses on efficiency calculations (B)	0.14
17	Effect of ^3^H *E_β_*_(max)_ on efficiency calculations (B)	0.09
18	Effect of ^63^Ni *E_β_*_(max)_ on efficiency calculations (B)	0.0024

**Relative combined standard uncertainty**		**0.40**

**Table 17:** NIST uncertainty analysis for the EUROMET intercomparison of ^55^Fe. The analysis makes no estimate for any uncertainty due to the possible presence of radionuclidic impurities

Item	Uncertainty component (of Type)	Relative standard uncertainty contribution tomassic activity of ^55^Fe (%)
1	LS measurement variability; reproducibility with 7 cocktails of comparable composition; *υ* = 6 degrees of freedom (A)	0.4
2	LS cocktail stability and composition effects; *υ* = 3 (A)	0.6
3	Gravimetric (mass) determinations for LS samples (B)	0.05
4	Background measurement variability; wholly embodied in item 1 above (A)	–
5	Spectrometer and scintillant dependencies; wholly embodied in items 1 and 2 above (A)	–
6	Livetime determinations for LS counting time intervals; includes uncorrected deadtime effects (B)	0.1
7	Decay corrections for ^55^Fe (B)	0.001
8	Experimental ^55^Fe efficiency from NIST ^55^Fe standard (B)	2.1
9	Variability in determination of QIPs; wholly embodied in items 1 and 10 (A)	–
10	Precision of fits for quench curves (efficiencyversus QIP) (A)	0.03

**Relative combined standard uncertainty**		**2.2**

**Table 18:** Results for the massic activity *C*_A_(^63^Ni) of the EUROMET ^63^Ni solution (in units of kBq · g^–1^ as reported by the various participating national metrological laboratories (as of the reference time 1200 UT 1 January 1996). The *C*_A_(^63^Ni) uncertainties correspond to the reported combined standard uncertainties

Laboratory	*C*_A_(^63^Ni)/(kBq · g^–1^)	Measurement method and comments
BIPM	39.9 ± 0.1	LS CNET; LPRI ^3^H standard; “BETA(11/94)” code
CIEMAT	39.96 ± 0.17	LS CNET; Amersham ^3^H standard; “EFFY5” code
CMI	41.77 ± 2.13	LS extrapolation technique
ENEA	40.28 ± 0.22	LS CNET; LPRI ^3^H standard; “EFFY4” code
IRMM	40.1 ± 0.2	LS CNET; IRMM ^3^H standard; “EFFY4(11/84)” code
LPRI	39.8 ± 0.2	LS TDCR
NAC(1)	39.72 ± 0.20	LS TDCR
NAC(2)	39.44 ± 0.17	LS CNET; LPRI ^3^H standard; “EFFY4(2/89)” code
NIST	39.80 ± 0.16	LS CNET; NIST & LPRI ^3^H standards; “EFFY4(1/93)” code
PTB	40.22 ± 0.34	LS CNET; PTB ^3^H standard; “EFFY-PTB” code
RC	40.34 ± 0.27	LS TDCR
SCK/CEN	40 ± 1	LS comparative measurement; LPRI ^63^Ni standard

**Table 19:** Results for the massic activity *C*_A_(^55^Fe) of the EUROMET ^55^Fe solution (in units of kBq · g^–1^) as reported by the various participating national metrological laboratories (as of the reference time 1200 UT 1 January 1996). The *C*_A_(^55^Fe) uncertainties correspond to the reported combined standard uncertainties

Laboratory	*C*_A_(^63^Ni)/(kBq · g^–1^	Measurement method and comments
BIPM	47.40 ± 0.40	LS CNET; LPRI ^3^H standard “BETA(11/94)” & “EMI” codes
CIEMAT	52.89 ± 1.00	LS CNET; Amersham ^3^H standard “CEGA2(1988)” code
CMI	50.46 ± 2.52	LS extrapolation and proportional counting (calibrated with ^54^Mn, ^57^Co, ^65^Zn and ^51^Cr standards)
ENEA	50.60 ± 0.60	LS CNET; LPRI ^3^H standard “MINERVA” code
IRMM	50.20 ± 0.40	LS CNET; IRMM ^3^H standard; “EFFY4 (11/84)”, “EMI” & “BETA” codes
LPRI	51.00 ± 0.20	LS TDCR
NAC	52.71 ± 0.42	4*π* (e,X)–*γ* coincidence efficiency tracing; ^54^Mn standard
NIST	52.95 ± 1.18	LS comparative measurement; NIST ^55^Fe standard;
PTB	51.10 ± 0.35	LS CNET; PTB ^54^Mn standard; “EFFY-PTB” and “EMI” codes
RC	51.20 ± 0.47	LS TDCR

## Footnotes

1 Certain commercial equipment, instruments, or materials are identified in this paper to foster understanding. Such identification does not imply recommendation or endorsement by the National Institute of Standards and Technology, nor does it imply that the materials or equipment are the best available for the purpose.
